# X-Ray Equipped with Artificial Intelligence: Changing the COVID-19 Diagnostic Paradigm during the Pandemic

**DOI:** 10.1155/2021/9942873

**Published:** 2021-08-22

**Authors:** Mustafa Ghaderzadeh, Mehrad Aria, Farkhondeh Asadi

**Affiliations:** ^1^Student Research Committee, Department and Faculty of Health Information Technology and Ma School of Allied Medical Sciences, Shahid Beheshti University of Medical Sciences, Tehran, Iran; ^2^Department of Computer Engineering, Faculty of Electrical and Computer Engineering, Shiraz University, Shiraz, Iran; ^3^Department of Health Information Technology and Management, School of Allied Medical Sciences, Shahid Beheshti University of Medical Sciences, Tehran, Iran

## Abstract

**Purpose:**

Due to the excessive use of raw materials in diagnostic tools and equipment during the COVID-19 pandemic, there is a dire need for cheaper and more effective methods in the healthcare system. With the development of artificial intelligence (AI) methods in medical sciences as low-cost and safer diagnostic methods, researchers have turned their attention to the use of imaging tools with AI that have fewer complications for patients and reduce the consumption of healthcare resources. Despite its limitations, X-ray is suggested as the first-line diagnostic modality for detecting and screening COVID-19 cases.

**Method:**

This systematic review assessed the current state of AI applications and the performance of algorithms in X-ray image analysis. The search strategy yielded 322 results from four databases and google scholar, 60 of which met the inclusion criteria. The performance statistics included the area under the receiver operating characteristics (AUC) curve, accuracy, sensitivity, and specificity.

**Result:**

The average sensitivity and specificity of CXR equipped with AI algorithms for COVID-19 diagnosis were >96% (83%-100%) and 92% (80%-100%), respectively. For common X-ray methods in COVID-19 detection, these values were 0.56 (95% CI 0.51-0.60) and 0.60 (95% CI 0.54-0.65), respectively. AI has substantially improved the diagnostic performance of X-rays in COVID-19.

**Conclusion:**

X-rays equipped with AI can serve as a tool to screen the cases requiring CT scans. The use of this tool does not waste time or impose extra costs, has minimal complications, and can thus decrease or remove unnecessary CT slices and other healthcare resources.

## 1. Introduction

Upon the emergence of COVID-19, the World Health Organization (WHO) designated the outbreak as a global health emergency and changed the disease state from an epidemic to a pandemic. One year has elapsed since that date, and on March 2, 2021, more than 120 million confirmed cases of COVID-19 have been reported globally. Of these, more than 2.65 million cases died [1]. The symptoms of COVID-19 are nonspecific; based on reports, in individuals and families with asymptomatic infections, CT scans show pneumonia, and the virus pathogenicity test is positive [2, 3]. To stop the spread of COVID-19 and decrease its mortality rate, early detection and effective screening of patients are urgent needs. The gold standard detection method for testing COVID-19 patients is RT-PCR (Reverse Transcription-Polymerase Chain Reaction) testing on respiratory specimens. This test is the most common method for detecting COVID-19, but it has disadvantages such as being manual, complicated, laborious, and time-consuming, and its positive rate is only 63%. In addition, during the pandemic, the low sensitivity of RT-PCR is not acceptable. As a result, infected people may not be identified and promptly treated and, due to the contagious nature of COVID-19, can spread the virus to healthy people [4, 5]. These shortcomings have encouraged healthcare specialists to present an alternative method with high efficiency for the detection and diagnosis of COVID-19. Since the beginning of 2020, based on clinical and paraclinical features of COVID-19, researchers have employed chest radiology modalities as effective tools for detecting, quantifying, and following-up COVID-19 cases. The indicators of infection include abnormalities in the patients' chest CT and X-ray images [6, 7]. In COVID-19 diagnosis, CT scans are more sensitive and specific than chest X-rays, and in many cases, lung involvement and GGO (Ground Glass Opocity) can be observed on CT scans even before the onset of clinical symptoms and a positive PCR test. However, problems such as high cost and the risk of spreading the disease when using the CT scan equipment may cause serious complications for patients and the healthcare system. Based on the American College of Radiology recommendation, CT scans should not be used as a first-line diagnostic modality. Since COVID-19 attacks the epithelial cells of the respiratory tract, specialists often use X-ray images to check the strength of the lungs and diagnose any kind of lung disease. Nevertheless, X-ray findings ranging from GGO to consolidations overlap other types of pneumonia [6–8]. It seems that, as the pandemic progresses, the medical community will often rely on CXR (Chest X-ray) due to its widespread availability and fewer infection control issues that currently limit the use of CT. Therefore, many researchers have utilized the X-ray modality to detect, diagnose, and classify COVID-19. However, the shortcomings of this imaging method have been mentioned in several texts [9–11]. Accordingly, many researchers turned to AI methods to address these deficiencies. Much effort has been made to develop AI-based medical systems based on the advances of digital image processing, pattern recognition, and computer vision. Such systems are expected to overcome the operator dependency, increase diagnosis efficiency rates, and reduce the need for medical complementary modalities [12, 13].

The present systematic reviews are aimed at introducing the latest technologies discussed in the COVID-19 literature which focuses on AI technologies for detecting/diagnosing the affected areas of the lungs. Instead of merely presenting a brief summary of the included studies, different statistical analyses by using graphs have been performed on various aspects of the system discussed in the selected papers. We present the following article in accordance with the PRISMA reporting checklist.

## 2. Method

### 2.1. Search Criteria

This systematic review aimed to identify various studies related to COVID-19 detection based on radiological images and AI classifiers. Specifically, it sought to answer the following research questions:

1. To what extent can the use of AI algorithms improve the common methods of COVID-19 diagnosis?
2. Which AI method is the most effective in analyzing COVID-19 X-ray images?

A systematic review was conducted to identify all the published studies in which AI algorithms had been utilized to detect/classify the X-ray images of suspected COVID-19 cases. Several electronic databases, including Scopus (http://www.scopus.com), Elsevier ScienceDirect (http://www.sciencedirect.com), PubMed (http://www.pubmed.ncbi.nlm.nih.gov), and Web of Science (http://www.wosg.ir), were searched from 2020 to January 2021. The following search keywords were used: “COVID-19,” “X-ray,” “artificial intelligence,” “machine learning,” “deep learning,” “detection,” “classification,” “computer-aided diagnosis,” “computer-aided detection,” and “computer-aided diagnosis.” The largest possible number of publications was investigated; still, some related studies may have been accidentally ignored.

### 2.2. Eligibility Criteria

The following are the eligibility criteria:

1. Being written in English
2. Using AI techniques to detect GGO and consolidation in COVID-19 patients
3. Examining COVID-19 detection and diagnosis based on X-ray images
4. Using AI algorithms for X-ray image analysis

### 2.3. Data Extraction

**Figure 1:**
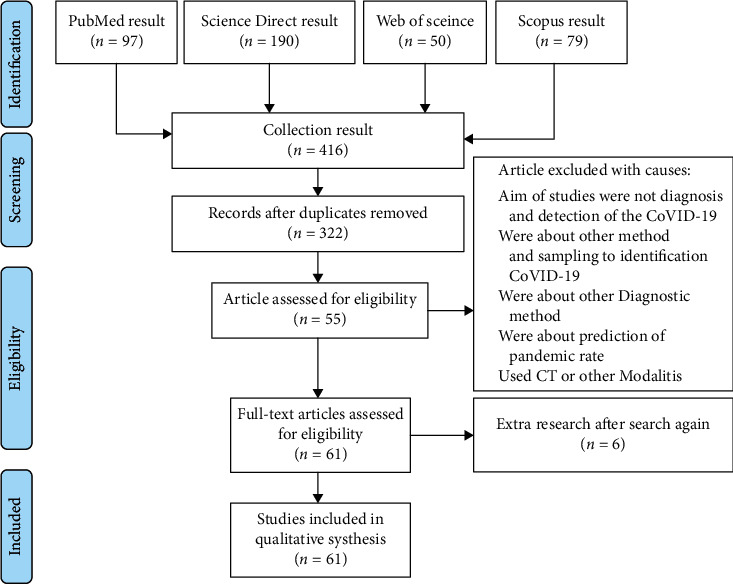
PRISMA flow diagram depicting the selection process for inclusion and exclusion.

Two authors (FA and MG) independently extracted the data and, after modifying the Cochrane standardized data extraction table based on the research questions, used it to assess the risk of bias for each study. The forms filled out by each author were compared, and the differences were resolved through researching, analysis, and discussion with the senior author as the final arbitrator. For predictive models, the data were extracted from the CHARMS checklist modified for the purpose of this study, which also includes an assessment of the risk of deviation [14]. This checklist is designed to evaluate all major predictive modeling research, including ANN (Artificial Neural Networks) and other types of ML (machine learning). After duplicate removal, 269 studies were identified. Next, 66 potentially relevant studies were selected by title/abstract screening, of which 60 remained after the full-text screening.  Figure 1 displays the PRISMA flowchart that summarizes the study selection procedure [15]. Note that many articles contained more than one AI algorithm, and they were all counted when forming this diagram.

## 3. Result

For each article, the data were extracted regarding (i) the country of the author team, (ii) the aim of the research, (iii) data volume, (iv) feature engineering, (v) AI methods and algorithms used, and (vi) efficiency.

Due to the wide range of AI algorithms, the studies had different ideas for using these algorithms to analyze X-ray images. Many of the studies were binary, meaning they dealt with only two classes of COVID-19 patients and non-COVID-19 patients. But some researchers have processed data from more than two data classes using machine learning concepts. Their data class included patients with COVID-19, patients with various types of pneumonia, people suspected of having COVID-19 but without lung involvement and completely healthy.

Based on the initial review of the relevant research, it was found that the AI methods were based on two techniques. The first technique is the application of traditional ML algorithms, and the second methodology was the utilization of DL algorithms for X-ray image analysis.

A number of studies utilized the classical concepts of ML [16–20] and even compared the performance of these classical algorithms with DL (deep learning) algorithms for COVID-19 diagnosis and classification [20, 21]. Many of them employed hybrid methods and more than one algorithm for processing and classifying the COVID-19 data; however, in many of them, several models and architectures were compared, and the model with the highest efficiency was extracted from these comparisons [22–26]. In an overview of the studies, almost all of these studies used pretrained networks. The use of these networks stems from a concept called transitional learning.

### 3.1. Transfer Learning

A concept that has received attention in many studies is transfer learning, according to which the knowledge extracted from large datasets is extracted by deep learning methods and is then transferred to a smaller but related dataset [27]. In the models employed to detect and classify COVID-19 using X-rays, the values of the efficient hyperparameters are transferred from the processed state of the art to the current problem [28, 29]. This is because large datasets are required to process the X-ray images of COVID-19 with the deep learning method, and such datasets do not exist. Therefore, pretrained models to apply the concept of transfer learning are used [30–40].

Shibly et al. and Zhang et al. altered the structure of these efficient pretrained architectures, which eventually led to better results in COVID-19 classification and diagnosis. Many researchers developed new models by developing pretrained models, which led to excellent results [33, 41–43]. Furthermore, in some of these studies, the aggregation of several pretrained networks, models, and techniques is used to perform high-quality feature extraction. By combining several well-known and efficient models, these studies provide the best performance of feature engineering [33, 44].

Based on the used X-ray datasets, several studies differentiated the data into two classes of patients with COVID-19 and non-COVID-19 patients [21, 24, 25, 29, 36, 39, 40, 42, 45, 46]. In others, the database included more than two classes, e.g., viral pneumonia, bacterial pneumonia, and normal and COVID-19 cases [17, 23, 30–35, 37, 39, 41, 43, 47–50]. Through the synthesis of the data, four domains of AI applications in X-ray analysis were identified:

### 3.2. AI Application Domain in COVID-19 Chest X-Ray Image Analysis

Through data synthesis, four applications of artificial intelligence in the analysis of X-ray images of the chest of people suspected of COVID-19 were identified. Detection (diagnosis), classification, lesion visualization, and segmentation and detection are four categorizations that had been used frequently in studies. Many studies have not distinguished semantic, lexical, and practical differences between these terminologies. The categorization made in this four areas is based solely on the terms used in the text of the study. The purpose of many studies was to combine several categories to achieve applied analysis.

#### 3.2.1. Detection and Diagnosis

Upon examining the existing texts and lexicons and seeking advice from radiologists and epidemiologists, *detection* is defined as part of the real entity that can be seen or whose existence can be proved or rejected. In medical texts, *detection* is considered a prelude to *diagnosis*. Cases have been identified in many studies aiming at identifying COVID-19 and its initial impact on lung tissue in its early stages. In these studies, the main purpose is to use early chest X-ray results to identify infection cases from other suspicious or normal cases. Twenty-eight studies aiming for detection used a spectrum of ML techniques for COVID-19 identification by X-ray image analysis [21, 22, 25, 27–49]. Another term that is very similar in function to *detection* is *diagnosis*. Although the two terms differ in clinical application, they are used interchangeably in various studies. Upon distinguishing these two terms from each other, *detection* is considered distinguishing the cases infected with COVID-19 and cases not infected with COVID-19; this means that there is no information about the class of non-COVID-19, and the group can have different types of bacterial pneumonia, viruses, or other coronavirus diseases, except for COVID-19. On the other hand, *diagnosis* distinguishes COVID-19 from other infectious lung diseases (e.g., different types of pneumonia) [17, 21, 24, 31, 34, 39, 40, 61, 62].

#### 3.2.2. Classification

Image classification is one of the earliest fields where ML has made a significant contribution to medical image analysis. Since the introduction and development of ML methods, numerous studies have adopted them for disease classification. In the field of radiological image analysis, considerable research has been conducted in the past years with the aim of classification. The main purpose of research aiming at classifying COVID-19 is to differentiate it from other diseases such as pneumonia by introducing an ML-based classification. In these studies, classification is performed to diagnose and detect COVID-19. In the literature, there are 27 studies for classification purposes, and COVID-19 infections are classified from other types of pneumonia and lung diseases. In such research, GGO and consolidation regions are classified from other suspected regions [18, 20, 55–59].

#### 3.2.3. Lesion Visualization

Object classification usually focuses on classifying a small, previously determined part of a medical image into two or more classes (e.g., nodule classification in lung X-ray). For many of these tasks, local information about the appearance of the lesion and global contextual information about the location of the lesion is required for accurate classification. To diagnose and classify COVID-19, many studies have extracted and displayed lung regions via radiographic images and used AI technology for this purpose. In these studies which mainly use the attention technique, the infected lungs are approximately shown. Some researchers who employed the X-ray technology to diagnose COVID-19 performed object detection, where the object was a lesion caused by COVID-19, showing the visual processing of the affected area of the lung. The difference between this and the segmentation method is that segmentation cannot transparently show the boundary of the lesion, and it roughly separates these areas from the texture. Attention map and heat map techniques were also employed to visualize CXR images so that the GGO region could be easily displayed using these technologies [23, 31, 37, 39, 44, 47, 54, 66, 67].

#### 3.2.4. Segmentation and Detection

Lung segmentation and COVID-19 disease are the removal of irrelevant regions on the image of lung tissue or the removal of normal areas of the lung, which play an important role in diagnosing diseases and displaying abnormal parts. Some studies have used other techniques, such as bounding boxes, to display and diagnose a healthy lung from an infected lung. Numerous studies have shown that segmentation, as one of the steps before COVID-19 classification, increases efficiency in disease detection. Watershed as a most popular segmentation technique is a transformation performed on grayscale images, used to segment different areas on the basis of geological watersheds to separate adjacent watersheds. It is like a topographic map, where the brightness of each point represents its height, and then find the line through the top of the ridge. In medical image segmentation, the watershed algorithm provides a complete division to separate meaningful feature regions for diagnosis [22, 60–65].

**Figure 2:**
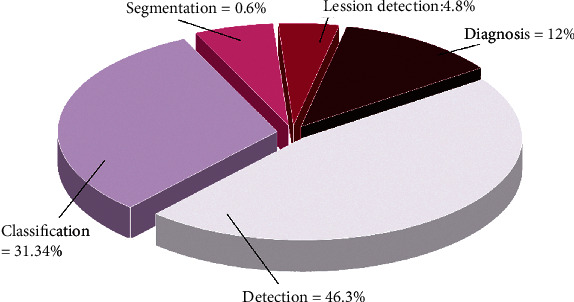
X-ray image analysis objectives.

Researchers have analyzed radiographic images to achieve one of these goals. The extent to which these goals have been attained in the literature is presented in Figure 2.

Since the 1950s, computer scientists have made efforts in the field of ML; however, in recent years, there has been a revolution in AI leading to the emergence of DL. As a subset of ML, DL is an end-to-end procedure whereby feature extraction is performed completely automatically [66].

In these methods, the building blocks of convolutional neural networks (including convolution and pooling layers) process the values corresponding to pixels. In this way, features can be automatically extracted. Then, the features are classified by feeding them into a layer containing one or more classifiers. These methods extract important features while ignoring secondary features. A review of research demonstrates that, to extract and process radiological image features for COVID-19 detection, many studies have adopted DL methods and algorithms and their latest models. Since researchers are dealing with radiological images in the diagnosis of COVID-19, and the volume of image data is very large; DL methods, especially CNN algorithms, yield better results. Based on the review of research on the COVID-19 diagnosis since its inception, many studies have utilized various DL algorithms to extract the features of radiological images. In all of these studies, DL methods have been employed to extract the features, and these features have been automatically extracted by CNN algorithms.

**Figure 3:**
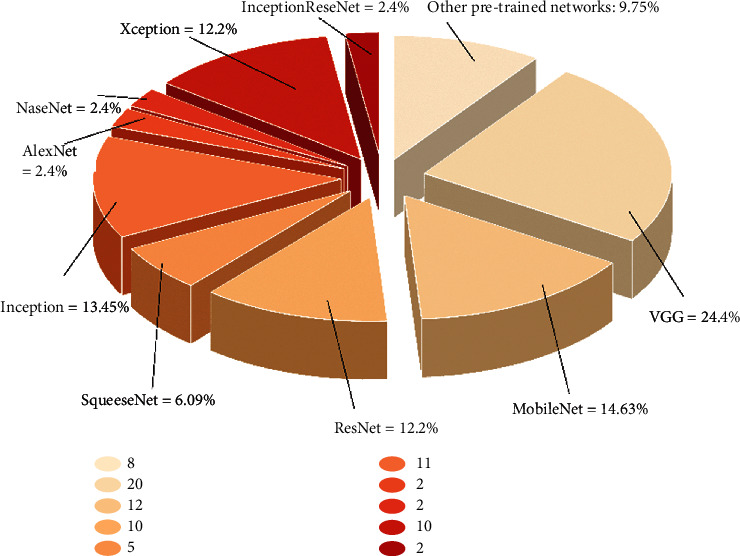
Rate of CNN pretrained structures in analyzing X-ray images.

In terms of effectiveness, one of the main characteristics of deep neural networks is the architecture they adopt. Some deep neural network architectures demonstrate an extraordinary ability to perform multiple functions for multiple data types. Various studies have been conducted on COVID-19 with different DL architectures, in which the diagnosis rate when detecting COVID-19 is compared by using several types of architectures (El Asnaoui & Chawki, 2020). The prevalent CNN architectures used in these studies can be seen in Figure 3. The CNN architectures have shown high performance in COVID-19 diagnosis based on CXR, and their performance differed based on the types of architecture and the predetermined model. Figure 3 illustrates the rate of use of these architectures. In the reviewed articles, the VGGNet architecture has played the greatest role. Nevertheless, some studies with different VGG16 architectures have achieved the best results in COVID-19 detection and diagnosis, while other studies have utilized other VGG19 versions to maximize the efficiency of analyzing radiological images for COVID-19 diagnosis. Newer and more developed architectures are found to be more effective in diagnosing COVID-19.

The list of the included articles and the most relevant characteristics and findings are presented in Table 1, including detection, diagnostic, and classification studies, in which in several studies the lesion visualization are presented in their research.

## 4. Discussion

This study reviewed the role of AI techniques in analyzing CXR images of COVID-19 suspected cases and described the employed algorithms by critically reviewing their performances. This systematic review presented 60 articles published on AI to improve the results of radiographic image analysis and lead to a more accurate diagnose, locate the affected lung area (GGO), and enhance the visual image of the lung of suspected COVID-19 cases.

### 4.1. A Review of the Shortcomings of These Studies

Despite the effectiveness of artificial intelligence methods in detecting COVID-19 using X-ray images, these methods have drawbacks and shortcomings that researchers have used techniques to escape these defects. Disadvantages of these methods include lack of balance datasets and lack of sufficient data for machine learning-based research.

#### 4.1.1. X-Ray Dataset Limitation

Due to the emergence of COVID-19 disease and the lack of bulk and suitable datasets for the applications of artificial intelligence, the researchers resorted to published datasets. But these datasets had variations in image angles, a limited number of images from different classes, and sometimes low-quality X-ray images. On the other hand, most artificial intelligence methods that input images, such as deep neural networks, depend on the size and number of images, and the larger the number of images and the size of the dataset, the better the results in image analysis. Numerous studies have shown that a small number of images lead to poor generalization and over fitting. Therefore, to solve this problem, many studies have used the data augmentation technique. The augmentation technique artificially inflates the training dataset size by either data warping or oversampling. Data augmentations transform existing images such that their label is preserved. This encompasses augmentations such as geometric and color transformations, random erasing, adversarial training, and neural style transfer. Oversampling augmentations create synthetic instances and add them to the training set. The augmentation technique artificially inflates the training dataset size by either data warping or oversampling. Data augmentations transform existing images such that their label is preserved. This encompasses augmentations such as geometric and color transformations, random erasing, adversarial training, and neural style transfer. Oversampling augmentations create synthetic instances and add them to the training set. Due to the limited data on COVID-19, almost all studies have used the data augmentation technique [25, 30, 31, 43, 45, 64, 89–91].

#### 4.1.2. Imbalance X-Ray Datasets

In many studies, while dealing with real datasets, they face the fundamental problem of nonclass balance distribution. Classifiers usually solve the problem to minimize global errors. These classifiers are more likely to consider majority classes when dealing with unbalanced datasets. Therefore, finding the wrong patterns will lead to incorrect labels. In the case of medical data and diseases, this imbalance is enormous. Studies have shown that this imbalance of data is the predominant problem in the emerging disease of COVID-19, which has occurred in almost all studies. Examining the datasets used in the studies, it can be seen that the data were unbalanced and included a smaller number of cases. And this reduced the sensitivity in diagnosing cases. Also, in studies that had a higher ratio of noninfected cases to infected cases, it led to a decrease in the specificity rate in diagnosing noninfected cases. In the real world, it is known that the number of cases of pneumonia and pulmonary disease is higher than the number of cases of COVID-19 and imbalance dataset COVID-19 X-ray images cause problems for research validation.

In order to solve the problem of unbalanced classification datasets, several methods have been proposed in the literature, and data-level solutions are the most famous and commonly used technology. The main goal of these techniques is to rebalance the class distribution by resampling the dataset to reduce the impact of class imbalance, that is, preprocessing the dataset before the training phase. One of the methods to solve the problem of data imbalance is the resampling method. Resampling methods can be subdivided into two categories: oversampling and undersampling. Both are used to adjust the class distribution of the dataset, that is, the ratio between different classes in the dataset. In the undersampling method, in order to balance the distribution of samples, some instances are deleted from the majority class. In the oversampling technique, some instances of the minority class are copied or synthesized to balance the distribution of the classes. There are several methods for resampling.  Table 2 shows some of these methods.

### 4.2. Radiologist vs. Artificial Intelligence in X-Ray Image Analysis

In the context of a global pandemic, infections may spread widely in the community. So far, studies have only evaluated the imaging of confirmed infections. Lack of CT scan devices in some geographical locations, the time-consuming tests of these devices, and the side effects due to their high dose are all factors motivating the presentation of alternative tests such as CXR. Despite the extensive use of CXR for other abnormalities, its specificity and sensitivity in the diagnosis of COVID-19, and how imaging features correlate with severity, are still unknown. Not much research has been conducted on the efficiency of the X-ray modality for COVID-19 detection. It is believed that this lack of acceptance is due to the nature and the low resolution of the images. In this imaging method, the radiology dose is very low; the radiation dose in CXR is 30-70 times lower, and naturally, the image quality is not acceptable compared to CT scans [96–99].

In a study conducted in 2020 by examining the electronic health record of patients with COVID-19, by comparing the diagnostic performance metrics of the two modalities of CT scan and X-ray, the efficiency of these common radiological methods in diagnosing cases of COVID-19 was calculated. The study showed that the sensitivity of the CXR method in diagnosing cases with COVID-19 was 0.56%. In the present review study, by examining all the researches of the research community that used artificial intelligence methods with the aim of diagnosing and identifying COVID-19 disease, their performance was extracted with three criteria of Sensetivity, specificity, and Accuracy.  Table 3 shows the performance rate obtained from the analysis of CXR images with artificial intelligence versus manual methods and by an expert in radiography and CT scans. The findings indicated that the sensitivity of the CXR method in diagnosing COVID-19 cases was 0.56%. In the present systematic review, by examining all the studies utilizing AI methods for COVID-19 diagnosis and identification, their performance was extracted with three criteria of sensitivity, specificity, and accuracy.  Table 3 shows the performance rate obtained from the analysis of CXR images with AI in COVID-19 cases versus other detection methods used by specialists in X-ray, CT scans, and PCR.

The efficiency metrics of all 60 studies were extracted and surveyed. Based on the comparison of common methods for COVID-19 diagnosis such as lung CT, RT-PCR, and X-ray equipped with AI algorithms, it can be concluded that AI is a strong and acceptable method for improving the detection coefficient and reducing diagnostic error in X-ray images. Using these algorithms, the visual defects of X-ray images can be overcome, and a high degree of detection of the GGO in the lung can be achieved. Furthermore, by using AI algorithms such as CNN, the exact patterns of lung involvement caused by COVID-19 can be classified from other forms of pneumonia. Since X-ray images are image-oriented and AI algorithms deal with pixel values, the use of DL methods such as CNN in extracting image features leads to better results in extracting the involved areas. Based on the review of 60 studies, more than 97% of them employed various DL algorithms to extract the features of X-ray images.

## 5. Conclusions

The control of a pandemic depends on the speed of contagion, which, in turn, largely depends on the ability and speed for reliably identifying the infected patients (a low false-positive rate). Local authorities in every country are currently facing the problem of reducing transmission, limiting the excessive use of medical facilities, and the number of virus-related deaths. In the pandemic, the main problem is that nasal swabs are only performed on people who show symptoms. Therefore, people currently infected with COVID-19 who are asymptomatic cannot be detected unless there are special circumstances [31, 105]. As a result, researchers looked for a cheaper, more affordable method with fewer side effects and found the answer in the use of X-rays. Still, this method was riddled with visual problems, so AI specialists provided computers with the ability to analyze X-ray images via learning-based solutions. AI provides an accurate and fast interpretation of complex data in large amounts and overcomes possible human error and/or bias. This progressively developing method can learn and gain experience and will continue to increase its success in accurate decision-making in the future. A wider application of AI in medical and infectious disease detection improves medical imaging interpretation, avoids wasting healthcare resources, and ultimately enhances the quality of patient care and outcome. The researchers suggest that a model based on deep learning algorithms should be implemented and developed in radiography (X-ray). In the entry of this model, all people are suspected of having COVID-19. If the proposed model confirms COVID-19 using X-ray images of the suspect, the physician will be advised to refer the patient for a CT scan or molecular test for further examination. During the COVID-19 pandemic, due to the great availability of radiographic devices, the X-ray method equipped with AI can be available in all healthcare centers to perform continuous and periodic testing of the entire community. This wide coverage will bring about a faster diagnosis and decrease the use of healthcare resources.

## Figures and Tables

**Table 1:** Original research studies that applied AI methods to analysis X-ray images of suspected to COVID-19 which met inclusion criteria.

Authors & country	Aim of study	Dataset description	Feature engineering	AI method	Model (structure)	Diagnostic performance
Saiz and Barandiaran (Spain) [45]	Detection	1600 image (204 COVID-19, 205 normal, 204 pneumonia for training, 100 COVID-19 images, 444 normal, 443 pneumonia images for testing)	Automatic	CNN using TL^1^	Vgg-16 and SDD^2^	Accuracy: 94.92% Sensitivity: 94.92% Specificity: 92%
Apostolopoulos et al. (Greece) [55]	Automatic detection	2870 image (224 COVID-19, 714 bacterial and viral pneumonias and 504 normal cases)	Automatic	CNN using TL	Mobilenet V2	Accuracy: 96.78% Sensitivity: 98.66% Specificity: 96.46%
Khan et al. (India) [44]	Detection and diagnosis	1251 images from four classes (310 normal, 330 bacterial pneumonia, 327 viral pneumonia, 284 COVID-19)	Automatic	Deep learning (Coronet)	Xception	Accuracy: 89.6%
Toğaçar et al. (Turkey) [39]	Detection	458 images (295 COVID-19, 98 pneumonia, and 65 normal)	Automatic	Deep learning and SVM	Squeezenet	Classification rate: 99.27%
Vaid et al. (Canada) [46]	Detection	108 images (34 COVID-19 and 75 normal)	Automatic	Deep learning (CNN)	VGG19	Accuracy: 96.3%
Rajaraman and Antani (USA) [27]	Detection	Four public datasets (detail was not mentioned)	Not mentioned	Deep learning (CNN)	Vgg-16	Sensitivity: 97.11% Specificity: 86.49% Accuracy: 93.08%
Yousri et al. (Egypt) [67]	Diagnosis	2 databases (216 COVID-19, 1675 non-COVID-19 in first dataset, and 219 COVID-19 and 1341 negative cases)	Frmems^3^	Deep learning via KNN	Mobilenet	Accuracy of first dataset: 96.09% Accuracy of second dataset: 98.09%
Apostolopoulos et al. (Greece) [28]	Detection	455 (detail not mentioned)	Automatic	Deep learning (CNN)	Mobilenet V2	Sensitivity: 97.36% Specificity: 99.42% Accuracy: 99.18%
Brunese et al. (Italy) [68]	Detection (differentiate)	6,523 (250 COVID-19, 2753 pulmonary diseases, 3520 normal)	Automatic	Deep learning (CNN)	Vgg-16	Accuracy: 97%
Pereira et al. (Brazil) [69]	Diagnosis (classification)	1144 (1000 normal, 90 COVID-19, 10 MERS, 11 SARS, 10 Varicella, 12 Streptococcus, and 11 Pneumocystis)	Automatic	Different algorithms	Inception-V3	F1 score: 89%
Ozturk et al. (Turkey) [48]	Automated detection	86 images (63 COVID-19, 6 Streptococcus, 11 SARS, 4 ARDS, 2 pneumocystis)	Automatic	Deep learning (CNN)	Darknet	Binary case accuracy: 98.08% Multiclass case accuracy: 87.02%
Ucar et al. (Turkey) [23]	Diagnosis	5949 images (1583 normal, 4290 pneumonia, and 76 COVID-19)	Automatic	CNN	Deep Bayes-SqueezeNet	Accuracy for overall class: 98.3%
Mahmud et al. (Bangladesh) [29]	Detection	6161 images (1583 normal, 1493 non-COVID-19 viral pneumonia, 2780 bacterial pneumonia, and 305 COVID-19 cases)	Not mentioned	Deep learning (CNN)	Convxnet	Accuracy of multiclass: 90.2%
Waheed et al. (India) [30]	Classification	1124 images (403 COVID-19 and 721 normal cases)	Automatic	Gan (COVID Gan)	Acgan^4^, Vgg16	Accuracy: 95% Sensitivity: 90% Specificity: 97%
El Asnaoui et al. (Moroco) [70]	Automatic detection	6087 (2780 bacterial pneumonia, 1493 COVID-19, 1583 normal)	Automatic	CNN	Inception_ResNet_v2	Acuracy: 92.18%
Sethy et al. (India) [6]	Detection	381 (127 COVID-19, 127 pneumonia, and 127 normal)	Automatic	CNN and SVM	Resnet50	Sensitivity: 95.33%
Das et al. (India) [51]	Screening (diagnosis)	6839 images (162 COVID-19, 5863 pneumonia, 814 TB^5^)	Automatic	CNN	Truncated Inception Net	Sensitivity: 88% Specificity: 100%
Martínez et al. (Columbia) [31]	Automatic detection	240 images (120 COVID-19 and 120 normal)	Automatic	CNN	Nasnet^6^	Accuracy: 97%
Yi et al. (USA) [71]	Classification (detection)	88 images (detail not mentioned)	Not mentioned	Deep learning	Not mentioned	Sensitivity: 89%
Loey et al. (Egypt) [72]	Detection (classification)	306 images (69 COVID-19, 79 normal, 79 bacterial pneumonia, and 79 viral pneumonia)	Automatic	Deep transfer learning and GAN	AlexNet GoogleNet ResNet	AlexNet testing accuracy: 85.2% GoogleNet testing accuracy: 100
Panwar et al. (India) [73]	Detection	337 images (192 COVID-19 and 145 non-COVID-19 pneumonia)	Automatic	Deep learning (Ncovnet)	Vgg16	Sensitivity: 97.62% Specificity: 78.57% Accuracy: 88.10%
Horry et al. (Australia) [37]	Detection (classification)	60798 images (115 COVID, 322 pneumonia, 60361 normal)	Automatic	CNN	VGG19	X-ray precision: 86% Ultrasound precision: 100% CT precision: 84%
Turkoglu (Turkey) [33]	Detection (classification)	6092 images (219 COVID-19, 1583 normal, and 4290 pneumonia)	Relief feature selection	SVM	Alexnet	Accuracy: 99.18%
Heidari et al. (USA) [74]	Detection classification	8474 images (415 COVID-19, 2880 normal, and 5179 pneumonias)	Automatic	Transfer learning-based CNN	VGG16	Accuracy: 94.5% Sensitivity: 98.4% Specificity: 98.0%
Tabik et al. (Spain) [75]	Classification	Normal-PCR+: 76, mild: 100, moderate: 171, severe: 79	Automatic	Deep learning (CNN)	Resnet-50	Accuracy: 97.72% ± 0.95%, 86.90% ± 3.20%, and 61.80% ± 5.49% in severe, moderate, and mild COVID-19 severity levels
Murugan and Goel (India) [22]	Classification	2700 images (900 images for each class; COVID, normal, pneumonia)	Automatic	Extreme learning machine classifier (ELM)	Resnet-50	Accuracy: 94.07 Sensitivity: 98.15 Specificity: 91.48
Ohata et al. (Brazil) [24]	Classification	Two dataset (194 COVID and 194 normal in each dataset)	Automatic	SVM-linear kernel (Dataset1)-MLP (Dataset2)	Mobilenet `(Dataset1) Densenet201 (Dataset2)	Dataset1 F1 score: 98.5 Dataset2 F1 score: 95.6
Mohammadi et al. (Iran) [25]	Detection	545 images (181 COVID-19 and 364 normal)	Automatic	Deep transfer learning CNN	(VGG)-16, VGG-19, Mobilenet, and Inceptionresnetv2	Acc of all model > 90% MobilenetAcc: 99.1% VGG16 AUC: 0.92% VGG19 AUC: 0.91% Mobilenet AUC: 0.99% Inceptionresnetv2: 0.97%
Narayan et al. (India) [38]	Detection	86 images (63 COVID-19, 6 Streptococcus, 11 SARS, 4 ARDS, 2 pneumocystis)	Automatic	Deep transfer	Inception (Xception)	Accuracy: 0.97% *F*-measure: 0.96%
Fan et al. (China) [76]	Detection	188 images (94 COVID-19 and 94 normal)	Automatic	Transfer learning CNN	Alexnet, Mobilenetv2, Shufflenet, Squeezenet, and Xception	Mobilenet average accuracy, recall, precision, and *F*-score: 97%, 96.5%, 97.5%, and 97%, respectively
Albadr et al. (Malaysia) [16]	Detection	188 images (two class including normal and COVID-19 cases)	Histogram of oriented gradients (HOG)	Optimized genetic algorithm-extreme learning machine	Not mentioned	Accuracy: 100.00% Recall: 100.00% *F*-measure (100.00%) and *G*-mean (100.00%)
Hussain et al. (Bangladesh) [56]	Detection classification	7390 images (2843 COVID-19, 3108 normal, pneumonias 1439) for 2 class, 3 class, and 4 class datasets	Automatic	CNN	Not mentioned	Classification accuracy For 2 class: 99.1% For 3 class: 94.2% For 4 class: 91.2%
Zhang et al. (China) [43]	Detection	5860 images (1585 normal and 4275 pneumonia)	Automatic	Transfer learning Deep residual network	Resnet-34	Accuracy: 91%
Khuzani et al. (USA) [18]	Classification	420 images (140 normal, 140 COVID-19, and 140 pneumonia)	Texture, FFT, wavelet, GLCM^7^, GLDM^8^	MLP	Not mentioned	Sensitivity: 100% Precision: 96%
Moujahid et al. (Morocco) [59]	Classification	5856 images (1583 normal and4273 pneumonia)	Automatic	CNN	VGG19	Precision: 96% Recal: 99% F1 score: 98% Accuracy: 96.58%
Afshar et al. (Canada) [52]	Classification	13,975 COVIDx dataset	Automatic	Capsule networks	Capsnets	Accuracy: 95.7% Sensitivity: 90% Specificity: 95.8% AUC: 97%
Dorr et al. (Argentina) [34]	Classification	302 images (102 COVID-19, 100 pneumonia, 100 normal)	Automatic	CNN	Densenet 121	Validation AUC: 0.96 External test AUC: 83%
Shorfuzzaman and Masud (Saudi Arabia) [77]	Classification	678 (226 COVID-19, 226 pneumonia and 226 normal)	Automatic	Deep Siamese Network	VGG-16ResNet50-V2 MobileNet, Xception, DenseNet121	ResNet50-V2: 98.06 MobileNet accuracy: 97.83%
Panahi et al. (Iran) [41]	Detection	940 images (435 COVID-19 and 505 non-COVID-19)	Automatic	CNN	Not mentioned	Accuracy: 96% F1-score: 96% AUC: 0.95%
Jain et al. (Germany) [35]	Classification detection	1215 (315 normal, 350 viral pneumonia, 300 bacterial pneumonia, and 250 COVID-19)	Automatic	Transfer learning with CNN	Resnet 50 Resnet-101	Accuracy: 98.93% Sensitivity: 98.93% Specificity: 98.66% F1-score: 98.15%
Shibly et al. (Bangladesh) [54]	Detection	232 images (283 COVID-19, 9501 non-COVID pneumonia, and 9466 normal)	Automatic	CNN	VGG-16	Accuracy: 97.36% Sensitivity: 97.65% Precision: 99.28%
Gupta et al. (India) [57]	Classification	3047 images (361 COVID-19, 1345 pneumonia, 1341 normal)	Automatic	Transfer learning CNN	Resnet101, Xception, Inceptionv3, Mobilenet, Nasnet	Accuracy: 99.08% Recall: 0.99% F1 score: 0.99
Phankokkruad (Thailand) [78]	Classification	274 COVID-19 cases, 380 viral pneumonia, and 380 normal cases	Automatic	Transfer learning CNN	Xception VGG16 Inception-Resnet	Xception accuracy: 97.19% VGG16 accuracy: 95.42% Inception-Resnet accuracy: 93.87%
Jain et al. (India) [36]	Classification	6432 (in training phase, 1345 are normal, 490 are COVID, and 3632 is pneumonia; in the validation phase, 238 samples of a normal case, 86 COVID, and 641 of pneumonia)	Automatic	Transfer learning CNN	Inception V3, Xception, Resnext	Xception reaches the highest Accuracy: 97.97%
Tartaglione et al. (Italy) [79]	Classification Segmentation	4 datasets (COVID-Chest XRay: 287, CORDA: 447, ChestXRay: 5857 RSNA: 26684)	Automatic segmentation using U-Net	CNN	ResNet-18 ResNet-50 COVID-Net DenseNet-121	AUC ResNet-18: [0.59, 1]% AUC ResNet-50: [0.61, 0.9]% AUC COVID-Net: [0.55, 0.85]% AUC DenseNet-121: [0.53, 0.9]%
Saha et al. (Bangladesh) [26]	Detection	4600 images (2300 COVID-19, 2300 non-COVID-19)	CNN	RF^9^, SVM^10^, DT^11^, ADAboost	VGG16	Accuracy: 98.91% Recall: 97.82% F1-score: 98.89%
Mostafiz et al. (Bangladesh) [22]	Detection	4809 images (790 COVID-19, 1215 viral pneumonia, 1304 bacterial pneumonia, and 1500 normal)	Hybrid model DWT^12^ CNN	RF	mRMR^13^ with RFE^14^	Overall accuracy of more than 98.5%
Abraham and Nair (India) [80]	Detection (CAD)	950 (453 COVID-19 and 497 non-COVID-19)	Multi-CNN with CFS^15^	Deep learning	CNN and Bayesnet classifier	Accuracy: 97.44% AUC: 91.16%
Deng et al. (China) [81]	Detection (classification)	Two datasets, 6624 images (1980 normal and 4644 pneumonia)	Automatic	SVM CNN	Resnet-50, Inceptionresnet-v2, Xception, Vggnet-16	First dataset accuracy: 84% Second dataset accuracy: 75%
Varela-Santos and Melin (Mexico) [20]	Classification	593 (detail of the data is not mentioned)	Texture features (GLCM^16^)	Neural network Deep learning	FFNN, feature-based FFNN, CNN	The results were calculated based on different datasets and different methods based on AUC and accuracy
Chandra et al. (India) [82]	Detection	2088 images (696 normal, 696 pneumonia, and 696 COVID-19)	Automatic	CNN	Ensemble majority voting (SVM, DT, KNN, ANN, NB)	Phase-I accuracy: 98% Phase-II accuracy: 91.3%
Islam et al. (Bangladesh) [40]	Detection	4575 (1525 pneumonia, 1525 normal, and 1525 COVID-19 cases)	Automatic using CNN network	LSTM^17^	Ordinary network	Accuracy: 99.4% Specificity: 99.2% Sensitivity: 99.3%
Minaee et al. (USA) [83]	Detection	5000 images (combination of different datasets was used)	Automatic	Transfer learning	Resnet18, Resnet50, Squeezenet, Densenet-121	Sensitivity: 98% ± 3% Specificity: 90% (Models have a similar performance)
Ismael and Şengür (Iraq) [21]	Classification	561 images (361 COVID-19 and 200 normal)	CNN	SVM (kernel: linear, quadratic, cubic Gaussian)	Resnet18, Resnet50, Resnet101, VGG16, VGG19	ResNet50 + SVM: 95.7% Wavelet transform: 96% Shearlet transform: 99.28%
Wang et al. (China) [53]	Classification	1102 images (565 normal, 537 COVID-19)	Automatic	Decision tree, random forest, Adaboost, bagging, SVM	VGG16, Inceptionv3, Resnet50, Densenet121, Xception	Xception + SVM accuracy: 99.33% Xception + SVM sensitivity: 99.27% Xception + SVM specificity: 99.38%
Hussain et al. (Pakistan) [84]	Classification	558 images (130 COVID-19, 145 viral pneumonia, 145 bacterial pneumonia, and 138 normal)	GLCM^18^ MFEM^19^	XGB-L^20^, XGB-Tree^21^, CART^22^, KNN^23^, NB^24^	GLCM^25^	Accuracy for pairwise data class: 96.3%, 100%
Rahaman et al. (China) [85]	Classification	860 images (260 COVID-19, 300 healthy, and 300 pneumonia cases)	Automatic	Transfer learning CNN	VGG series, Xception, ResnetvResnetv2, Inception, Densenet, Mobilenet	Accuracy: 89.3% Average recall: 89% Average F1 score: 90%
Gomes et al. (Brazil) [19]	Classification	6039 images (453 COVID-19, 1490 viral pneumonia, 2783 bacterial pneumonia, and 1583 normal)	Haralick, Zernike moments	MLP, SVM, RF Bayesian networks NB RT^26^	Not mentioned	Average accuracy: 89.78% Average sensitivity: 89.79% Average specificity: 99.63%
Ozturk et al. (Turkey) [86]	Classification	1127 images (127 COVID-19, 500 normal, and 500 pneumonia)	Hand craft (GLCM, LBGLCM^27^, GLRLM^28^, and SFTA^29^)	Classical machine learning approach	SVM	Accuracy: 86.54% Sensitivity: 83.15% Specificity: 96.96%
Altan and Karasu (Turkey) [87]	Diagnosis	7980 images (2660 normal, 2660 COVID-19, and 2660 viral pneumonia)	Coefficients with CSSA^30^ optimization method	Classical machine learning approach	Swarm algorithm and deep learning	Accuracy: 99.69% Sensitivity: 99.41% Specificity: 99.81%
Tuncer et al. (Turkey) [88]	Detection	321 images (234 normal and 87 COVID-19)	ResExLBP^31^ for FE^32^ IRF^33^ for FS^34^	Classical machine learning approach	SVM	Accuracy: 100%

^1^Transfer learning. ^2^Single shot detector. ^3^Fractional Multichannel Exponent Moments (FrMEMs). ^4^Auxiliary Classifier Generative Adversarial Network. ^5^Tuberculosis. ^6^Neural Architecture Search Network. ^7^Gray-level cooccurrence matrix. ^8^Gray-level difference method. ^9^Random forest. ^10^Support vector machine. ^11^Decision tree. ^12^Discrete wavelet transform. ^13^Minimum redundancy and maximum relevance. ^14^Recursive feature elimination. ^15^Correlation-based feature selection. ^16^Gray-level cooccurrence matrix (GLCM). ^17^Long short-term memory. ^18^Grey-level cooccurrence matrix. ^19^Morphological feature-extracting method. ^20^XG boosting linear. ^21^XG boosting tree. ^22^Classification and regression tree. ^23^K-nearest neighbor. ^24^Naïve Bayes. ^25^Grey-level cooccurrence matrix. ^26^Random tree. ^27^local binary gray-level cooccurrence matrix. ^28^Gray level run length matrix. ^29^Segmentation-based fractal texture analysis. ^30^Chaotic salp swarm algorithm. ^31^Residual exemplar local binary pattern. ^32^Feature extraction. ^33^Iterative relief. ^34^Feature selection.

**Table 2:** Most of the most well-known methods of resembling.

Method	Objective	Main
SMOTE [92]	Oversampling	Creates synthetic samples by combining the existing ones
ADASYN [93]	Oversampling	Creates synthetic samples for the minority class adaptively
SMOTE-B1/B2 [94]	Oversampling	Creates synthetic samples considering the borderline between the classes
TomekLinks [95]	Undersampling	Removes samples which are the nearest neighbors but have different labels
ENN/RENN [95]	Undersampling	Removes samples in which its label differs from the most of its nearest neighbors
AllKNN [95]	Undersampling	Removes samples in which a kNN algorithm misclassifies them
SMOTE+TL [95]	Hybrid	Applies SMOTE and TomekLink algorithms

**Table 3:** Comparison of the diagnostic efficiency X-ray equipped with artificial intelligence methods with conventional methods in the analysis of COVID-19.

COVID-19 diagnosis method	Author	Sensitivity	Specificity
CT scan efficiency rate (by radiologist)	Borakati et al. [96]	0.85 (95% CI 0.79 to 0.90)	0.50 (95% CI 0.41 to 0.60)
	Ai et al. [100]	0.97 (95% CI 95% to 98%)	25%
	Kovács et al. [101]	(67%–100%)	(25%–80%)
	Himoto et al. [102]	97%	56%
RT-PCR efficiency rate	Ai et al. [100]	65%	83%
	Cheng et al. [103]	47%	100%
	Caruso et al. [104]	58%	96%
X-ray efficiency rate (by radiologist)	Borakati et al. [96]	0.56 (95% CI 0.51 to 0.60)	0.60 (95% CI 0.54 to 0.65)
X-ray equipped with AI methods efficiency rate	Present systematic review	Average > 97% (83%-100%)	Average > 93% (80%-100%)
